# Potential Exosome Biomarkers for Parkinson’s Disease Diagnosis: A Systematic Review and Meta-Analysis

**DOI:** 10.3390/ijms25105307

**Published:** 2024-05-13

**Authors:** Ka Young Kim, Ki Young Shin, Keun-A Chang

**Affiliations:** 1 Department of Nursing, College of Nursing, Gachon University, Incheon 21936, Republic of Korea; kykim@gachon.ac.kr; 2 Neuroscience Research Institute, Gachon University, Incheon 21565, Republic of Korea; 3 Bio-MAX Institute, Seoul National University, Seoul 08826, Republic of Korea; 4 Department of Pharmacology, College of Medicine, Gachon University, Incheon 21999, Republic of Korea

**Keywords:** Parkinson’s disease, blood biomarker, exosome, α-synuclein

## Abstract

Parkinson’s disease (PD) is the second most common neurodegenerative disease worldwide. Given its prevalence, reliable biomarkers for early diagnosis are required. Exosomal proteins within extracellular nanovesicles are promising candidates for diagnostic, screening, prognostic, and disease monitoring purposes in neurological diseases such as PD. This review aims to evaluate the potential of extracellular vesicle proteins or miRNAs as biomarkers for PD. A comprehensive literature search until January 2024 was conducted across multiple databases, including PubMed, EMBASE, Web of Science, and Cochrane Library, to identify relevant studies reporting exosome biomarkers in blood samples from PD patients. Out of 417 articles screened, 47 studies were selected for analysis. Among exosomal protein biomarkers, α-synuclein, tau, Amyloid β 1-42, and C-X-C motif chemokine ligand 12 (CXCL12) were identified as significant markers for PD. Concerning miRNA biomarkers, miRNA-24, miR-23b-3p, miR-195-3p, miR-29c, and mir-331-5p are promising across studies. α-synuclein exhibited increased levels in PD patients compared to control groups in twenty-one studies, while a decrease was observed in three studies. Our meta-analysis revealed a significant difference in total exosomal α-synuclein levels between PD patients and healthy controls (standardized mean difference [SMD] = 1.369, 95% confidence interval [CI] = 0.893 to 1.846, *p* < 0.001), although these results are limited by data availability. Furthermore, α-synuclein levels significantly differ between PD patients and healthy controls (SMD = 1.471, 95% CI = 0.941 to 2.002, *p* < 0.001). In conclusion, certain exosomal proteins and multiple miRNAs could serve as potential biomarkers for diagnosis, prognosis prediction, and assessment of disease progression in PD.

## 1. Introduction

Parkinson’s disease (PD) is the second most common neurodegenerative disorder after Alzheimer’s disease [1], affecting roughly 10 million people globally, with ~2% prevalence among those >80 years old [2]. PD is a disease in which parts of the brain are progressively damaged over several years, resulting in motor symptoms such as tremor, bradykinesia, rigidity, as well as physical and psychiatric symptoms such as depression, anxiety, anosmia, insomnia, memory problems, etc. [3]. The exact cause of PD remains largely unknown, with current research pointing to a mix of genetic and environmental factors [4]. PD is histologically characterized by the specific loss of dopamine-producing neurons, particularly in the substantia nigra pars compacta, accompanied by the presence of abnormal protein clumps called Lewy bodies (LB) and Lewy neurites, containing α-synuclein (α-syn) [5,6]. Lewy bodies are abnormal protein aggregates found inside neurons, primarily composed of alpha-synuclein, observed in neurodegenerative diseases such as PD. In contrast, Lewy neurites are abnormal accumulations of protein within the processes (neurites), predominantly containing alpha-synuclein as well. Both are indicative of neuronal dysfunction and degeneration, with Lewy neurites observed in the dendrites and axons of affected neurons, closely related to the formation of Lewy bodies.

Diagnosing PD, particularly in its early stages, presents significant challenges due to the lack of definitive diagnostic tests, resulting in confirmation in only 80–90% of cases post-mortem [7,8,9]. Initial diagnosis relies on hallmark “parkinsonism” symptoms, including slow movement, tremors, and stiffness, the first being crucial and requiring the presence of ≥1 of the other two symptoms [10]. While the aggregation and spread of toxic forms of α-syn are key features of PD, as a diverse disorder, it poses challenges for developing and identifying useful biomarkers for diagnosis and disease progression, as well as for successfully translating new treatments to the clinic [11]. Therefore, there is an urgent need for reliable biomarkers to improve clinical diagnoses, treatment response assessments, and disease progression monitoring [12]. Additionally, identifying minimally invasive, reliable, repeatable, and cost-effective blood-based biomarkers is crucial for PD, where diagnostic criteria rely on analyzing proteins in cerebrospinal fluid and clinical/imaging assessments [13].

Recently, extracellular vesicles (EVs) have been proposed as biomarkers for diagnosing and predicting chronic neurodegenerative diseases [14]. EVs, comprising exosomes (50–150 nm), microvesicles (100–1000 nm), and apoptotic bodies (up to 5 μm), have been investigated for their potential as biomarkers in various neurological disorders, including PD [13,15]. Advances in exosome purification methods, such as size exclusion chromatography, have highlighted their potential as biomarker carriers [16,17]. Further, as mediators of intercellular communication, exosomes have been implicated in transmitting misfolded proteins between neurons, which explains their potential as biomarkers [18]. EVs are notably rich in non-coding RNAs, such as miRNAs, lncRNAs, and circRNAs, exhibiting broad distribution throughout both the brain and peripheral systems. They serve as crucial mediators linking normal neuronal function with disease pathology [19]. Particularly noteworthy is their capacity to facilitate the transportation and delivery of various miRNAs, among which miR-21 stands out. While typically associated with microglial anti-inflammatory responses, miR-21 has also been implicated in inflammatory contexts and highlighted as a potential novel biomarker for PD [20]. Several key categories of exosome biomarkers have been identified for PD: (i) α-synuclein-related markers: alpha-synuclein, Lewy body, etc.; (ii) neurotransmitter-related markers: dopaminergic neuron, dopamine transporter, etc.; (iii) inflammation- and immune system-related markers: TNF alpha, cytokine, etc. [2]; (iv) Alzheimer’s disease-related markers: tau, presenilin, etc. [21]; (v) miRNA biomarkers: downregulation of miR-1, miR-22, and miR-29a, as well as the upregulation of miR-16-2, miR-26a-2, and miR-30a, in PD patients [22].

Therefore, our aim is to explore the potential of exosomal proteins and miRNAs as biomarkers for PD, and to evaluate a candidate exosomal protein in peripheral blood as a potential PD biomarker through systematic reviews and meta-analyses.

## 2. Materials and Methods

### 2.1. Literature Search Strategy

This review collected studies published until January 2024 by searching four databases including PubMed, Embase, Web of Science, and Cochrane Library. The review was performed in accordance with the Preferred Reporting Items for Systematic Reviews and Meta-Analyses (PRISMA) guidelines (http://www.prisma-statement.org) (accessed on 2 January 2024). The following keywords were used for this research: (exosome OR exosomal OR exosomes [MeSH]) AND (Parkinson OR Parkinson disease [MeSH]) AND (blood OR blood [MeSH] OR plasma OR plasma [MeSH] OR serum OR serum [MeSH]) AND (biomarker OR biomarkers [MeSH] OR “biological marker”). Two authors (KYK and KAC) independently conducted searches and extracted articles after analyzing the title, abstract, and full text. In case of discrepancies, all three authors engaged in discussions.

### 2.2. Inclusion and Exclusion Criteria

The PICOS (population, intervention, comparison, outcome, and study design) framework was used to define the eligibility criteria. The inclusion criteria were as follows. (1) Participants included all PD patients diagnosed with PD regardless of factors such as stage, or presence of dementia. (2) The interventions reviewed in this study included evaluations of protein or miRNA PD biomarkers in exosomes in blood samples. (3) The comparator to PD patients was healthy controls. (4) The outcome was assessed by the levels of biomarkers. (5) Study designs included randomized controlled trials, epidemiological observational studies including cross-sectional, case–control, and cohort studies. The exclusion criteria were as follows: (1) studies including patients with other diseases such as Alzheimer’s disease, multiple system atrophy, progressive supranuclear palsy, and rapid eye movement sleep behavior disorder; (2) studies including biomarkers analyzed in CSF, tissue, cell, or animal samples; (3) studies not including healthy controls; (4) studies not related to blood exosomes; studies not related to protein or miRNA biomarkers; and (5) studies in form of letter, editorial, commentary, conference abstract, or systematic, scoping, umbrella, or literature reviews.

### 2.3. Data Extraction and Analysis

From the selected final studies, the following data were extracted: authors and publication years, study country, sample size, sex distribution, or age in PD and control groups, sample characteristics, statistically significant biomarkers reported in the extracted papers, and origin of exosomes. For the meta-analysis, we examined the standardized mean difference (SMD) in total exosomal or neuron-derived exosomal α-syn levels between PD and healthy control groups using the Comprehensive Meta-Analysis software version 4 (Biostats Inc., Englewood, NJ, USA). We used a random-effects model following the examination of the Q statistic and *I*^2^ method to assess heterogeneity. Statistical significance was determined at a *p* < 0.05. Furthermore, the articles included in this review underwent quality assessment using the Critical Appraisal Skills Programme (CASP, Oxford, UK, https://casp-uk.net/casp-tools-checklists/, accessed on 2 January 2024) checklists for randomized controlled trial, case–control study, and cohort study. The CASP tool comprises 11 questions across three sections: “Are the results of the study valid? (Section A)”, “What are the results? (Section B)”, and “Will the results help locally? (Section C)”.

## 3. Results and Discussion

### 3.1. Literature Search

**Figure 1:**
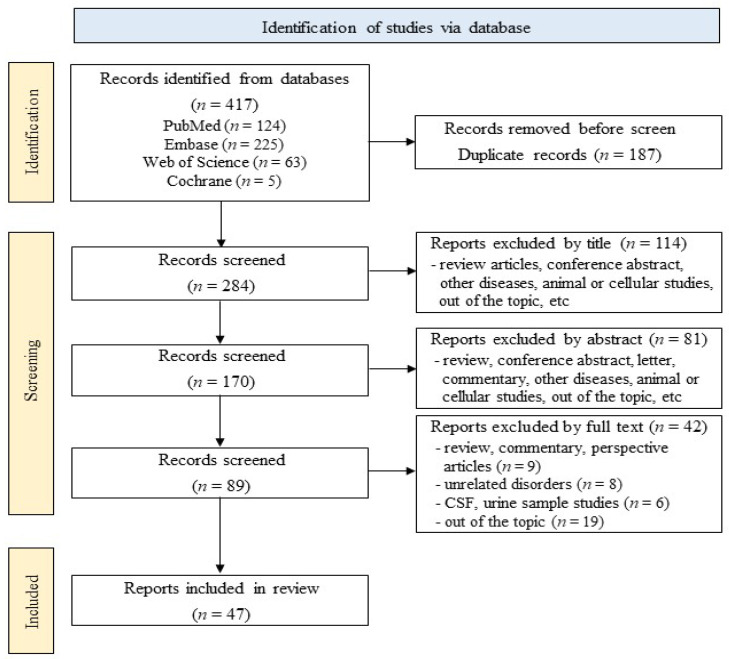
Flowchart of the literature search.

Figure 1 illustrates the process for literature search and selection. For this systematic review, a total of 417 articles, including 124 from PubMed, 225 from Embase, 63 from Web of Science, and 5 from the Cochrane Library, were screened according to the inclusion and exclusion criteria. Using EndNote and manual screening, 187 articles, including duplicates and irrelevant studies, were removed, resulting in 284 articles being extracted. Title analysis served to extract 170 articles, and abstract analysis to extract 89 articles. Finally, after a close review of the full text, 47 articles were used for this systematic review. These selected studies included blood exosomal biomarkers that showed significant changes in PD patients compared to healthy control groups, with significance determined based on the statistical significance presented in each paper. Furthermore, the quality assessment scores of the included studies were evaluated using the CAST checklist for case–control study, ranging from 5 to 10 points (Supplementary Table S1).

Table 1 and Table 2 present the general characteristics of the studies included in this systematic review. We included studies published between 2014 and 2023 and conducted in Italy Taiwan, China, USA, UK, Germany, New Zealand, Japan, South Korea, Turkey, and India. The study groups included PD patients and healthy controls. Sex and age are presented according to study group. Human blood samples included plasma and serum. Exosomes were derived from total, neuron, oligodendroglia, astrocyte, blood cell, epithelial cell, platelet, and vascular smooth muscle cells. Table 1 shows the characteristics of 34 exosomal protein biomarkers in PD, while Table 2 illustrates the characteristics of 14 exosomal miRNA biomarkers in PD. One study included both protein and miRNA biomarkers.

### 3.2. Potentially Important Exosomal Biomarkers in PD

Table 3 presents the exosomal PD biomarkers present in ≥2 studies among those considered significant in each study. Among them, α-syn levels increased in PD patients compared to the control group in twenty studies and decreased in two studies. Furthermore, tau, Amyloid β 1-42, and C-X-C motif chemokine ligand 12 (CXCL12) were identified as significant exosomal protein PD biomarkers in two studies.

First, plasma exosomal Aβ and tau are potential diagnostic candidates for PD. While the primary pathognomonic protein of PD is α-syn, other proteins like tau and Aβ have also been detected [66]. Clinicopathologic evidence suggests that a combined metric of LB, Aβ, and tau pathologies best correlates with dementia in PD [24,67,68]. The formation of α-syn oligomers leads to the generation of Aβ sheet fibrils, which aggregate into LB [13]. Cognitive dysfunction is a common nonmotor feature of PD, involving executive functions, attentional and visuospatial function, and memory. Approximately 15% of PD patients exhibit mild cognitive impairment at diagnosis [69], and many progress to dementia in the long term [70,71]. Early diagnosis and intervention are crucial for improving the prognosis and quality of life for PD patients with cognitive impairment [3]. Cognitive decline in PD results not only from the loss of dopaminergic neurons but also from involvement of serotonergic, glutaminergic, and cholinergic neurons in the subcortex and cortex [72]. Pathologically, cognitive dysfunction in PD is strongly associated with a combination of LB and Alzheimer’s pathology, involving both α-syn and Aβ [73,74]. Despite understanding the pathological roles of these proteins in PD, their application as biomarkers remains challenging. Studies have investigated the use of α-syn, Aβ, and tau as PD biomarkers [75].

Second, plasma exosomal CXCL12 can also be a potential diagnostic candidate for PD. Chemokines can be categorized into four subfamilies: the CXC subfamily, characterized by cysteine residues separated by a single amino acid; the CC chemokines, featuring two adjacent cysteine residues; the XC chemokines, which contain a single cysteine residue in the amino terminus; and the CX3C subfamily, with three amino acid residues separating the cysteine tandem [76]. The regulation of chemokine activity involves a network of feedback loops and mechanisms responsible for their suppression and/or stimulation [77]. The levels of chemokines in the extracellular fluid regulate inflammation, infection, immunological responses, tissue injury reactions, apoptosis, and immune cell trafficking [77,78]. Among homeostatic chemokines, CXCL12, also known as stromal-derived factor 1 (SDF-1), is one of the most evolutionarily conserved chemokines, binding to the CXCR4 receptor. CXCL12 and CXCR4 are widely expressed in the central nervous system (CNS), primarily on astrocytes and microglia in the normal CNS, as well as on neurons in the adult brain [79]. CXCL12 has attracted attention as a potential therapeutic target for promoting nerve regeneration, and emerging evidence suggests its involvement in regulating autophagy [80]. With respect to miRNAs, miRNA-24, miR-23b-3p, miR-195-3p, miR-29c, and mir-331-5p were identified as significant exosomal biomarkers in ≥2 studies. Plasma exosomal miRNAs (e.g., miR-24, miR-23b-3p, miR-29c, miR-195-3p, and miR-331-5p) stand as potential diagnostic candidates for PD. Within neurodegenerative disorders, the intricate pathophysiological landscape is notably influenced by miRNA gene regulation [81]. These small RNAs, typically around 22 nucleotides in length, exert post-transcriptional control over gene expression by forming base pairs with target mRNAs [82]. Evidence from multiple studies underscores the varied expression patterns of miRNAs within the human brain, some modulating genes implicated in neurodegeneration [83]. Specifically, exosomal miRNAs have garnered attention as promising circulating biomarkers due to their resilience against endogenous RNase degradation, stable presence, and detectability even in minute concentrations [84]. A wealth of research highlights the pivotal roles played by exosomal miRNAs in disease progression and their potential clinical utility as diagnostic indicators [58]. Noteworthy findings are as follows: (i) upregulation of miR-24 in PD patients, with potential associations to PD-like phenotypes [12,55,63]; (ii) identification of miR-23b-3p as a novel circulating miRNA linked to PD, known for its role in mitigating neuroinflammation and neuronal apoptosis [56,85,86]; (iii) significantly increased miR-29c expression in PD patients compared to controls [60]; (iv) miR-195 upregulation in PD patients [12,60,87]; and (v) elevated miR-331-5p expression in both serum exosomes and CSF exosomes of PD patients [63,64].

### 3.3. Meta-Analysis on Total Exosomal and Neuron-Derived Exosomal α-Synuclein

**Figure 2:**
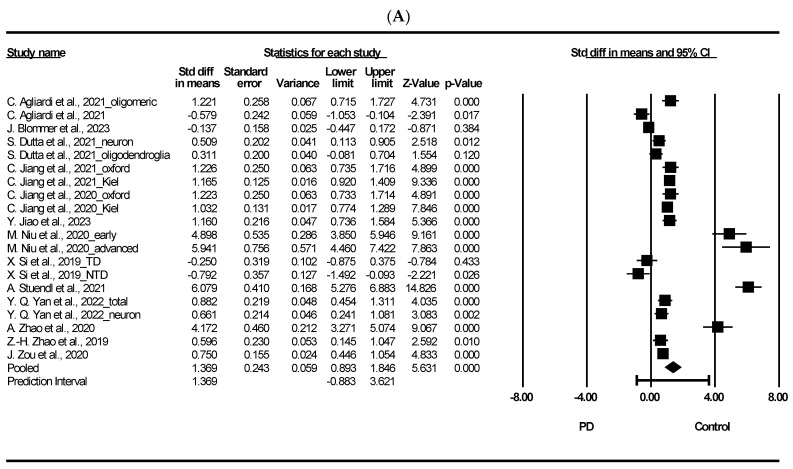

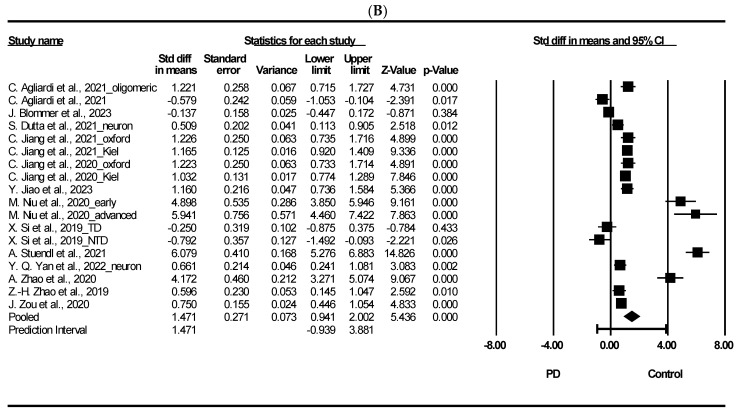
Forest plots of exosomal and neuron-derived exosomal α-synuclein. (**A**) Exosomal α-synuclein, (**B**) neuron-derived exosomal α-synuclein. Std diff: standard difference, CI: confidence interval. Individual study effect is represented by a square, while the pooled effect across studies is represented by a diamond [13,24,31,33,34,35,38,44,45,49,50,51,53].

Figure 2 presents the results of a meta-analysis on the potential of total exosomal and neuron-derived exosomal α-syn as exosomal biomarker in PD. As shown in Figure 2A, the meta-analysis of total exosomal α-syn showed that patients with PD had a significant difference to heathy controls (standardized mean difference [SMD] = 1.369, 95% confidence interval [CI] = 0.893 to 1.846, *p* < 0.001). Moreover, neuron-derived exosomal α-syn showed that PD had significant difference compared to healthy control (SMD = 1.471, 95% CI = 0.941 to 2.002, *p* < 0.001). We discuss the potential of plasma exosomal α-syn as a diagnostic tool for PD. Exosomes, nano-sized EVs, are released into the extracellular matrix by various cell types and are abundant in body fluids such as plasma, urine, and cerebrospinal fluid [88]. Their cargo can reflect the intracellular environment of the originating cells and participate in intracellular communication under different physiological and pathological conditions [89]. In PD, exosomes may accelerate α-syn aggregation; in fact, there is evidence on their involvement in disease progression via prion-like spread of pathogenic misfolded α-syn [90]. α-syn is a 140-amino-acid protein mainly found in the brain, particularly in presynaptic areas, although it is also detectable in the nucleus of brain cells and peripheral organs [91]. It regulates synaptic vesicle dynamics at nerve terminals and dopamine neurotransmission, directly influencing mitochondrial physiology, potentially linking mitochondrial dysfunction to PD pathogenesis [91,92]. Mitochondrial defects may contribute to LB formation, a pathological hallmark of PD [93]. α-syn is readily secreted into extracellular spaces, and its levels can be measured in cerebrospinal fluid (CSF), plasma/serum, red blood cells, and saliva [94]. Despite variations in total α-syn levels in PD [91], our findings indicate significantly higher levels of α-syn in PD patients compared to healthy controls. Additionally, several studies have demonstrated elevated levels of α-syn in plasma neuronal-derived exosomes from PD patients compared to healthy controls [32,38,51,95]. It was reported that the interaction between α-syn and membranes plays a significant role in the conformational shifts of α-syn, potentially impacting protein functionality and contributing to aggregation in PD progression [2]. Furthermore, the aggregated and harmful variant of the protein, oligomeric α-syn, has been observed to engage with lipids, inducing structural alterations in lipid membranes. This interaction leads to various effects, including membrane disruption, thinning, pore formation, and lipid clustering [96]. Additionally, oligomeric α-syn appears to be influenced by post-translational modifications (PTMs) like phosphorylation, nitration, and dopamine (DA) modification, as noted in studies [97].

## 4. Conclusions

Here, we reviewed the potential of exosomal proteins such as α-syn, amyloid-β (Aβ), tau, and C-X-C motif chemokine ligand 12 (CXCL12), as well as miRNAs (miR-24, miR-23b-3p, miR-29c, miR-195-3p, and miR-331-5p) as biomarkers for PD. A systematic review and meta-analysis suggest that α-syn could be an effective exosomal biomarker protein.

First, we conclude that plasma exosomal α-syn could serve as an effective biomarker for PD, because our findings indicate significantly higher levels of α-syn in PD patients compared to healthy controls. Second, we suggest that plasma exosomal Aβ and tau may also serve as effective biomarkers for PD, particularly concerning cognitive function, because it has been shown that the annual changes in plasma EV tau and Aβ1-42 levels significantly differ between PD patients and healthy controls [27]. Third, we conclude that plasma exosomal CXCL12 may serve as an effective biomarker for PD, because previous studies have indicated that peripheral blood levels of CXCL12 correlate with pathological processes and disease progression in PD, making them potential diagnostic and prognostic markers [98,99]. In addition, CXCL12 levels have been associated with nonmotor symptoms such as autonomic dysfunction, while α-syn levels in plasma neuronal exosomes have been linked to clinical stage, motor symptoms, and nonmotor symptoms [34]. Fourth, we suggest that plasma exosomal miRNAs, including miR-24, miR-23b-3p, miR-29c, miR-195-3p, and miR-331-5p, hold promise as effective biomarkers for PD, because circulating miRNAs contribute significantly to the pathogenesis of numerous chronic conditions, including PD [100]. However, this study has several limitations. First, our findings are constrained by the data sourced from the literature included in this study. Since our study was limited to including both PD and healthy control groups, certain exosomal biomarkers in PD patients may not have been fully represented. Additionally, the presence of repeated data from the same researcher could potentially introduce bias into the results. Second, our analysis included both control and PD groups without stratification by PD stage. While analyzing PD stages is crucial, it was challenging in this study due to many included studies not providing information on the stages of the subjects. Consequently, further research is warranted to examine the specific stages of PD. Third, while studies with various exosomal biomarkers were included in this research, studies with analyzable data from two or more sources were limited. Therefore, only α-syn was included in the meta-analysis. Continuous research is necessary for potential exosomal biomarkers. Nonetheless, the significant alterations observed in exosomal α-syn levels among PD patients underscore the potential of exosomal proteins such as Aβ, tau, and CXCL12, along with various miRNAs as promising biomarkers for PD.

## Figures and Tables

**Table 1:** General characteristics of studies on potential exosomal protein biomarkers.

Study (Author, Year)	Country	Sex (M/F or *N*)		Age (M ± SD)		Sample	Significant Biomarker	Exosome Origin
		PD	Control	PD	Control			
C. Agliardi et al., 2021 [13]	Italy	21/11	21/19	67.5 ± 7.6	69.5 ± 8.6	Serum	Oligomeric α-synuclein, STX-1A, VAMP-2	Neuron
F. Anastasi et al., 2021 [23]	Italy	4	4			Plasma	PSMA1-3, PSMA5-7, PSMB1, PSMB3, PSMB5-6, PARK7, Gelsolin, Amyloid P component, Clusterin, CXCL12	Neuron
J. Blommer et al., 2023 [24]	New Zealand	156/68	28/21	71.8 ± 7.0	75.8 ± 7.3	Plasma	α-synuclein, pTau 18, pY-IRS-1	Neuron
S. Cerri et al., 2018 [25]	Italy	25/14	15/18	65.2 ± 8.9	61.9 ± 6.2	Plasma	α-synuclein	
L. Chan et al., 2021 [26]	Taiwan	86/27	9/39	69.7 ± 8.4	67.9 ± 7.5	Plasma	TGF-b1, pro-IL-1β, TNF-α	
L. Chan et al., 2023 [27]	Taiwan	55/48	25/12	68.2 ± 10.0	66.6 ± 10.8	Plasma	Tau, Aβ1-42, α-synuclein	
Z. T. Chen et al., 2023 [28]	China	26/17	17/17	66.6 ± 9.6	63.7 ± 10.0	Plasma	ferritin	Neuron
S. Y. Chou et al., 2020 [29]	Taiwan	48/46	39/24	69.0 ± 8.2	68.0 ± 7.6	Plasma	p-IRS-1S312	Neuron
C. C. Chung et al., 2021 [30]	Taiwan	62/54	18/28	69.7 ± 8.4	67.0 ± 7.0	Plasma	Tau, Aβ1-42	
S. Dutta et al., 2021 [31]	USA	32/19	22/28	71.5 ± 9.5	63.2 ± 12.2	Plasma	α-synuclein	Neuron/Oligodendroglia
Y. Fu et al., 2020 [32]	UK	20	20			Serum	α-synuclein	Neuron
C. Jiang et al., 2021 [33]	UK	(Oxford) 36/12	22/9	62.8 ± 9.3	66.3 ± 8.8	Serum	α-synuclein	Neuron
		(Brescia) 17/10		65.0 ± 9.4				
		(Kiel) 136/79	72/41	67.6 ± 4.8	59.0 ± 4.8			
		(PROSPECT)	17/30		68.0 ± 6.8			
C. Jiang et al., 2020 [34]	UK	(Oxford) 36/12	22/9	62.8 ± 9.3	66.3 ± 8.8	Serum	α-synuclein	Neuron
		(Kiel) 96/59	72/41	67.5 ± 9.3	59.0 ± 4.8			
		(Brescia) 17/10		65.0 ± 9.4				
Y. Jiao et al., 2023 [35]	China	22/28	25/25	64.3 ± 5.6	64.0 ± 5.8	Plasma	CCL2, CXCL12, α-synuclein	
Y. Kitamura et al., 2018 [10]	Japan	5/3	5/3	63.5 ± 6.8	62.0 ± 5.8	Plasma	apolipoprotein A1, clusterin, complement C1r subcomponent, fibrinogen gamma chain	
A. Kluge et al., 2022 [36]	Germany	21/9	34/16	67 [46–84] ^a^		Plasma	α-synuclein	Neuron
B. Leng et al., 2020 [3]	China	11 (12)	11/9	65.0 ± 8.0	65.5 ± 5.0	Plasma	Prion	
F. Lucien et al., 2022 [37]	USA	57	20			Plasma	α-synuclein	
M. Niu et al., 2020 [38]	China	11/10	25/28	64 ± 5.4	65 ± 5.3	Plasma	α-synuclein	Neuron
A. Picca et al., 2020 [39]	Italy	7/9	7/5	74.5 ± 8.4	75.5 ± 4.9	Serum	CD9, CD63, ATP5A, NDUFS3, SDHB	
M. Sharafeldin et al., 2023 [40]	UK	20	20			Serum	α-synuclein, synt-1	Neuron
M. Shi et al., 2014 [41]	USA	145/119	116/99	66.3 ± 9.1	65.7 ± 9.1	Plasma	α-synuclein	Neuron
M. Shi et al., 2016 [42]	USA	65/26	58/48	65.0 ± 11.1	67.1 ± 7.4	Plasma	Tau	Neuron
K. H. Shim et al., 2021 [43]	South Korea	30/4	24/5	74.2 ± 4.7	73.9 ± 4.6	Plasma	acetylcholinesterase	Neuron, blood cell, epithelial cell
X. Si et al., 2019 [44]	China	(TD) 12/10	8/10	62.7 ± 10.6	62.7 ± 2.4	Serum	α-synuclein	Neuron
		(NTD) 9/9		62.1 ± 10.6				
A. Stuendl et al., 2021 [45]	Germany	96	42	65.0 ± 11.8	61.6 ± 14.1	Plasma	α-synuclein	Neuron
P. Wang et al., 2023 [46]	China	59/47	58/45	60.9 ± 13.9	56.5 ± 12.5	Plasma	α-synuclein	Astrocyte
Z. Wang et al., 2023 [47]	China	36/37	28/22	67.5 ± 8.2	62 [59,67] ^b^	Plasma	Aβ1-42	Platelet
S. Yan et al., 2024 [48]	UK	30/20	11/9	63.6 ± 6.1	61.3 ± 10.7	Serum	α-synuclein	Neuron
Y. Q. Yan et al., 2022 [49]	China	19/25	23/26	64.2 ± 9.6	61.5 ± 7.1	Plasma	t-exo α-synuclein, n-exo α-synuclein	Neuron, VSMC
A. Zhao et al., 2020 [50]	China	17/13	15/15	62.3 ± 8.5	61.4 ± 9.1	Plasma	α-synuclein	
Z.-H. Zhao et al., 2019 [51]	China	23/16	17/23	67.5 ± 6.9	66.6 ± 8.8	Plasma	α-synuclein, DJ-1	Neuron
H. Zheng et al., 2021 [52]	China	19/17	17/19	70.4 ± 0.6	69.2 ± 0.4	Plasma	Oligomer/total α-synuclein	Neuron
J. Zou et al., 2020 [53]	China	53/40	48/37	66.9 ± 9.5	66.2 ± 10.3	Plasma	α-synuclein, Linc-POU3F3	Neuron

^a^, Mean [range]; ^b^, Mean [median], PD, Parkinson’s disease; STX-1A, Syntaxin 1A; VAMP-2, Vesicle-Associated Membrane Protein 2; PSMA1-3, Proteasome subunit alpha 1-3; PSMA5-7, Proteasome subunit alpha 5-7; PSMB1, Proteasome subunit beta 1; PSMB3, Proteasome subunit beta 3; PSMB5-6, Proteasome subunit beta 5-6; PARK7, Parkinsonism-associated deglycase; Aβ, Amyloid β; CXCL12, C-X-C motif chemokine ligand 12; CCL2, C-C motif chemokine ligand 2; ATP5A, Adenosinetriphosphate5A; NDUFS3, NADH: ubiquinone oxidoreductase subunit S3; SDHB, Succinate dehydrogenase complex iron sulfur subunit B; VSMC, vascular smooth muscle cell; TD, tremor dominant; NTD, non-tremor dominant; t-exo, total exosomal; n-exo, neural-derived exosomal; POU3F3, POU class 3 homebox 3.

**Table 2:** General characteristics of studies on potential exosomal miRNA biomarkers.

Study (Author, Year)	Country	Gender (M/F or *N*)		Age (M ± SD)		Sample	Significant Biomarker	Exosome Origin
		PD	Control	PD	Control			
M. A. Aguilar et al. 2023 [54]	USA	33/27	20/20	66.3 ± 10.9	66.6 ± 9.9	Serum	miR-26b-5p, RNA5SP382, piR_009295, piR_020498, piR_020492, miR-181a-5p, piR_016658, miR-25-3p, miR-191-5p, piR_004153, p-hsa_miR-330, miR-6073, miR-221-3p, miR-21-5p, piR_004150, RNA5SP259_RNA5SP25, RNU6-1300P, piR_019825, piR_015068, RNA5-8SP6, RNA5-8SP4, piR_002468, RNA5SP485, piR_022606, piR_004152, RNA5SP253_RNA5SP26, piR_005019, piR_017754	Neuron
F. Anastasi et al. 2021 [23]	Italy	4	4			Plasma	miR-155	Neuron
C. Barbagallo et al. 2020 [55]	Italy	24/6	10/20	69.6 ± 8.0	67.9 ± 8.2	Serum	let-7d, miR-22, miR-23a, miR-24, miR-142-3p, miR-222	
M. Cai et al. 2021 [56]	China	7	5			Plasma	miR-23b-3p, miR-30b-5p, miR-195-3p, miR-195-5p	
X. Y. Cao et al. 2017 [12]	China	73/36	25/15	69.8 ± 9.2	67.9 ± 8.6	Serum	miR-195, miR-24, miR-19b	
L. A. Citterio et al. 2023 [57]	Italy	26/19	25/24	67.3 ± 9.0	65.5 ± 12.2	Serum	miR-223-3p	
I. Manna et al. 2021 [58]	Italy	26/19	15/24	66.4 ± 8.6	63.7 ± 7.5	Serum	miR-21-3p, miR-223-5p, miR-22-3p	
C. Nie et al., 2020 [59]	China	1/6	10/10	61.9 ± 8.4	34.0 ± 11.5	Plasma	miR-197-3p, miR-576-5p, miR-1468-5p, miR-375, let-7e-5p, miR-211-5p, let-7e-3p, miR-122-3p, miR-941, miR-30d-5p, miR-192-5p, miR-93-5p, miR-425-5p, miR-99b-5p, let-7i-5p, miR-652-3p, miR-4732-3p, miR-6131, miR-3184-3p, miR-378g	Neuron
B. Ozdilek et al., 2021 [60]	Turkey	31/20	12/8	64.3 ± 8.8	58.6 ± 7.1	Serum	miR-29c	
S. Rai et al., 2023 [61]	India	13/3	13/3	55.6 ± 14.2	55.2 ± 12.7	Plasma	miR-23b-3p	
D. Sproviero et al., 2021 [62]	Italy	9	6	69 ± 3.6	55 ± 5.2	Plasma	miR-6509-5p, miR-1266-5p, miR-30c-2-3p, miR-4646-5p, miR-195-3p, miR-4442, miR-7161-3p, miR-1262, miR-5089-5p, miR-4433b-5p, miR-4451, miR-4778-5p, miR-4286, miR-30a-3p, miR-6068, miR-3152-3p, miR-485-3p, miR-6728-5p, miR-4642, miR-579-5p, miR-3614-3p, miR-574-5p, miR-520a-3p, miR-4657, miR-4740-3p, miR-660-3p, miR-5001-5p, miR-3184-5p, miR-7856-5p, miR-365a-5p, miR-1-3p, miR-1275, miR-433-3p, miR-767-3p	
G. Tong et al., 2022 [63]	China	115/94	29/21	68.2 ± 5.4	65.6 ± 4.3	Serum	miR-151a-5p, miR-24, miR-485-5p, miR-331-5p, mir-214, miR-29b-2-5p, miR-29c, miR-16-2-3p, let-7d-5p, miR-200a, miR-126, miR-221, miR-148b, miR-19a, miR-29b, miR-126a, miR-151-5p, miR-24, miR-374a, miR-15b, let-7a, miR-29a-3p, miR-626, miR-301a, miR-28-5p, miR-1, miR-29c	
Y.-F. Yao et al., 2018 [64]	China	52	48	65.6 ± 10.5	61.2 ± 9.0	Plasma	miR-331-5p, miR-505	
X. Zhang et al., 2017 [65]	China	22/24	22/27	63.1 ± 1.5	60.35 ± 1.2	Plasma	miR-433, miR-133b	

**Table 3:** Potentially important exosomal biomarkers in PD.

Exosomal Biomarkers	Variation	Studies Suggested to be Significant	No. of Overlap
α-synuclein	Increase	C. Agliardi et al., 2021 [13], S. Cerri et al., 2018 [25], L. Chan et al., 2023 [27], S. Dutta et al., 2021 [31], Y. Fu et al., 2020 [32], C. Jiang et al., 2021 [33], C. Jiang et al., 2020 [34], Y. Jiao et al., 2023 [35], A. Kluge et al., 2022 [36], F. Lucien et al., 2022 [37], M. Niu et al., 2020 [38], M. Sharafeldin et al., 2023 [40], M. Shi et al., 2014 [41], A. Stuendl et al., 2021 [45], P. Wang et al., 2023 [46], S. Yan et al., 2024 [48], Y. Q. Yan et al., 2022 [49], A. Zhao et al., 2020 [50], Z.-H. Zhao et al., 2019 [51], J. Zou et al., 2020 [53]	20
	Decrease	J. Blommer et al., 2023 [24], X. Si et al., 2019 [44]	2
tau	Increase	L. Chan et al., 2023 [27], M. Shi et al., 2016 [42]	2
Aβ 1-42	Increase	L. Chan et al., 2023 [27], Z. Wang et al., 2023 [47]	2
CXCL12	Increase	F. Anastasi et al., 2021 [23], Y. Jiao et al., 2023 [35]	2
miR-24	Increase	C. Barbagallo et al., 2020 [55], X. Y. Cao et al., 2017 [12], G. Tong et al., 2022 [63]	3
miR-23b-3p	Increase	S. Rai et al., 2023 [61]	1
	Decrease	M. Cai et al., 2021 [56]	1
miR-195-3p	Increase	M. Cai et al., 2021 [56], D. Sproviero et al., 2021 [62]	2
miR-29c	Increase	B. Ozdilek et al., 2020 [60], G. Tong et al., 2022 [63]	2
mir-331-5p	Increase	G. Tong et al., 2022 [63], Y.-F. YAO et al., 2018 [64]	2

PD, Parkinson’s disease; Aβ, Amyloid β; CXCL12, C-X-C motif chemokine ligand 12.

## Data Availability

The data that support the findings of this study are available from the corresponding authors upon reasonable request.

## Supplementary Materials

The following supporting information can be downloaded at: https://www.mdpi.com/article/10.3390/ijms25105307/s1.
