# Supplementary material for: Potential Exosome Biomarkers for Parkinson’s Disease Diagnosis: A Systematic Review and Meta-Analysis
Source: Int J Mol Sci. 2024 May 13;25(10):5307. doi: 10.3390/ijms25105307 (PMC11121363; doi:10.3390/ijms25105307)
Supplement: Supplementary file 1 [file ijms-25-05307-s001.zip › ijms-2956171-supplementary.pdf]

Supplementary Table S1. Quality assessment using CASP checklist.

Study	1	2	3	4	5	6	7	8	9	10	11	Score out of 11
C. Agliardi et al., 2021	Y	Y	Y	Y	Y	Y	significant (p<0.001)	Precise (95% CI used)	Y	?	Y	10
M. A. Aguilar et al. 2023	Y	Y	N	Y	Y	Y	significant (FDR<0.05)	can't tell	Y	?	Y	8
F. Anastasi et al., 2021	Y	Y	Y	Y	Y	Y	significant (p<0.05)	can't tell	Y	?	Y	9
C. Barbagallo et al. 2020	Y	Y	Y	Y	Y	?	significant (p<0.05)	Precise (95% CI used)	Y	?	Y	9
J. Blommer et al., 2023	Y	Y	Y	Y	Y	?	significant (p<0.05)	can't tell	Y	?	Y	8
M. Cai et al. 2021	Y	Y	Y	Y	Y	?	significant (p<0.05)	can't tell	Y	?	Y	8
X. Y. Cao et al. 2017	Y	Y	Y	Y	Y	?	significant (p<0.001)	can't tell	Y	?	Y	8
S. Cerri et al., 2018	Y	Y	Y	Y	Y	Y	significant (p<0.001)	can't tell	Y	?	Y	9
L. Chan et al., 2021	Y	Y	Y	Y	Y	Y	significant (p<0.05)	Precise (95% CI used)	Y	?	Y	10
L. Chan et al., 2023	Y	Y	Y	Y	Y	Y	significant (p<0.05)	can't tell	Y	?	Y	9
Z. T. Chen et al., 2023	Y	Y	Y	Y	Y	?	significant (p<0.05)	Precise (95% CI used)	Y	?	Y	9
S. Y. Chou et al., 2020	Y	Y	Y	Y	Y	?	significant (p<0.05)	Precise (95% CI used)	Y	?	Y	9
C. C. Chung et al., 2021	Y	Y	Y	Y	Y	Y	can't tell	can't tell	Y	?	Y	8
L. A. Citterio et al. 2023	Y	Y	Y	Y	Y	Y	significant (p<0.001)	can't tell	Y	?	Y	9
S. Dutta et al., 2021	Y	Y	Y	Y	Y	?	significant (p<0.05)	can't tell	Y	?	Y	8
Y. Fu et al., 2020	Y	Y	Y	Y	Y	?	significant (p<0.001)	can't tell	Y	?	Y	8
C. Jiang et al., 2021	Y	Y	Y	Y	Y	?	can't tell	can't tell	Y	?	Y	7
C. Jiang et al., 2020	Y	Y	Y	Y	Y	?	significant (p<0.001)	can't tell	Y	?	Y	8
Y. Jiao et al., 2023	Y	Y	Y	Y	Y	Y	significant (p<0.05)	Precise (95% CI used)	Y	?	Y	10
Y. Kitamura et al., 2018	Y	Y	Y	Y	Y	Y	significant (p<0.05)	can't tell	Y	?	Y	9
A. Kluge et al., 2022	Y	Y	Y	Y	Y	?	significant (p<0.001)	can't tell	Y	?	Y	8
B. Leng et al., 2020	Y	Y	Y	Y	Y	Y	significant (p<0.05)	can't tell	Y	?	Y	9
F. Lucien et al., 2022	Y	Y	Y	Y	Y	Y	significant (p<0.001)	can't tell	Y	?	Y	9
I. Manna et al. 2021	Y	Y	Y	Y	Y	?	significant (p<0.05)	can't tell	Y	?	Y	8
C. Nie et al., 2020	Y	Y	Y	?	Y	?	significant (p<0.05)	can't tell	Y	?	?	6
M. Niu et al., 2020	Y	Y	Y	Y	Y	?	significant (p<0.05)	Precise (95% CI used)	Y	?	Y	9
B. Ozdilek et al., 2021	Y	Y	?	Y	Y	Y	significant (p<0.05)	Precise (95% CI used)	Y	?	Y	9
A. Picca et al., 2020	Y	Y	Y	Y	Y	Y	significant (p<0.05)	can't tell	Y	?	?	8
S. Rai et al., 2023	Y	Y	?	Y	Y	Y	significant (p<0.05)	can't tell	Y	?	Y	8
M. Sharafeldin et al., 2023	Y	Y	?	?	Y	?	significant (p<0.05)	can't tell	Y	?	Y	6
M. Shi et al., 2014	Y	Y	Y	Y	Y	Y	significant (p<0.05)	can't tell	Y	?	Y	9
M. Shi et al., 2016	Y	Y	Y	Y	Y	Y	significant (p<0.05)	can't tell	Y	?	Y	9

K. H. Shim et al., 2021	Y	Y	Y	Y	Y	Y	significant (p<0.05)	can't tell	Y	?	?	8
X. Si et al., 2019	Y	Y	Y	Y	Y	Y	significant (p<0.05)	can't tell	Y	?	Y	9
D. Sproviero et al., 2021	Y	Y	Y	Y	Y	Y	can't tell	can't tell	Y	?	Y	8
A. Stuendl et al., 2021	Y	Y	?	?	Y	?	significant (p<0.05)	can't tell	Y	?	?	5
G. Tong et al., 2022	Y	Y	Y	Y	Y	?	significant (p<0.05)	can't tell	Y	?	Y	8
P. Wang et al., 2023	Y	Y	Y	Y	Y	?	significant (p<0.05)	can't tell	Y	?	Y	8
Z. Wang et al., 2023	Y	Y	?	?	Y	?	significant (p<0.05)	can't tell	Y	?	Y	6
S. Yan et al., 2024	Y	Y	Y	?	Y	?	significant (p<0.05)	can't tell	Y	?	Y	7
Y. Q. Yan et al., 2022	Y	Y	Y	Y	Y	Y	significant (p<0.05)	can't tell	Y	?	Y	9
Y.-F. Yao et al., 2018	Y	Y	Y	?	Y	?	significant (p<0.05)	can't tell	Y	?	Y	7
X. Zhang et al., 2017	Y	Y	?	?	Y	?	significant (p<0.05)	can't tell	Y	?	?	5
A. Zhao et al., 2020	Y	Y	Y	?	Y	Y	significant (p<0.05)	can't tell	Y	?	Y	8
Z.-H. Zhao et al., 2019	Y	Y	?	Y	Y	?	significant (p<0.05)	can't tell	Y	?	Y	7
H. Zheng et al., 2021	Y	Y	Y	Y	Y	?	significant (p<0.05)	can't tell	Y	?	Y	8
J. Zou et al., 2020	Y	Y	Y	?	Y	?	significant (p<0.05)	can't tell	Y	?	Y	7

1. Did the study address a clearly focused issue? 2. Did the authors use an appropriate method to answer their question? 3. Were the cases recruited in an acceptable way? 4. Were the controls selected in an acceptable way? 5. Was the exposure accurately measured to minimize bias? 6. (a) Have the authors taken account of the potential confounding factors in the design and/or in their analysis? 7. How large was the treatment effect? 8. How precise was the estimate of the treatment effect? 9. Do you believe the results? 10. Can the results be applied to the local population? 11. Do the results of this study fit when other available evidence?

Y: Yes, ?: can't tell, CI: confidence interval
